# Structure-based discovery of inhibitors of the YycG histidine kinase: New chemical leads to combat *Staphylococcus epidermidis *infections

**DOI:** 10.1186/1471-2180-6-96

**Published:** 2006-11-10

**Authors:** Zhiqiang Qin, Jian Zhang, Bin Xu, Lili Chen, Yang Wu, Xiaomei Yang, Xu Shen, Soeren Molin, Antoine Danchin, Hualiang Jiang, Di Qu

**Affiliations:** 1 Key laboratory of Medical Molecular Virology of Ministry of Education and Ministry of Pulic Health, Institute of Medical Microbiology and Institutes of Biomedical Sciences, Shanghai Medical School of Fudan University Box 228, Yi Xue Yuan Road 138#, Shanghai 200032, PR China; 2 Drug Discovery and Design Center, State Key Laboratory of Drug Research, Shanghai Institute of Materia Medica, Graduate School of Chinese Academy of Sciences, Shanghai Institutes for Biological Science, Chinese Academy of Sciences, 65 Zuchongzhi Road, Shanghai 201203, PR China; 3 Infection Microbiology Group, BioCentrum-DTU, Technical University of Denmark, DK-2800 Lyngby, Denmark; 4 Genetics of Bacterial Genomes, CNRS URA 2171, Institut Pasteur, 28 rue du Docteur Roux, 75724, Paris Cedex 15, France

## Abstract

**Background:**

Coagulase-negative *Staphylococcus epidermidis *has become a major frequent cause of infections in relation to the use of implanted medical devices. The pathogenicity of *S. epidermidis *has been attributed to its capacity to form biofilms on surfaces of medical devices, which greatly increases its resistance to many conventional antibiotics and often results in chronic infection. It has an urgent need to design novel antibiotics against staphylococci infections, especially those can kill cells embedded in biofilm.

**Results:**

In this report, a series of novel inhibitors of the histidine kinase (HK) YycG protein of *S. epidermidis *were discovered first using structure-based virtual screening (SBVS) from a small molecular lead-compound library, followed by experimental validation. Of the 76 candidates derived by SBVS targeting of the homolog model of the YycG HATPase_c domain of *S. epidermidis*, seven compounds displayed significant activity in inhibiting *S. epidermidis *growth. Furthermore, five of them displayed bactericidal effects on both planktonic and biofilm cells of *S. epidermidis*. Except for one, the compounds were found to bind to the YycG protein and to inhibit its auto-phosphorylation *in vitro*, indicating that they are potential inhibitors of the YycG/YycF two-component system (TCS), which is essential in *S. epidermidis*. Importantly, all these compounds did not affect the stability of mammalian cells nor hemolytic activities at the concentrations used in our study.

**Conclusion:**

These novel inhibitors of YycG histidine kinase thus are of potential value as leads for developing new antibiotics against infecting staphylococci. The structure-based virtual screening (SBVS) technology can be widely used in screening potential inhibitors of other bacterial TCSs, since it is more rapid and efficacious than traditional screening technology.

## Background

In recent years, coagulase-negative strains of *Staphylococcus epidermidis *have become frequent causes of infections in connection with surgically implanted medical devices [1,2]. In parallel, the appearance of multi-resistant and vancomycin-resistant *S. epidermidis *strains has increased quickly due to the increasing use of antibiotics in hospitals [3]. The primary pathogenicity trait of *S. epidermidis *has been associated with its ability to form biofilms on surfaces of medical devices, limiting severely the efficacy of many conventional antibiotics, and biofilms may also protect the bacteria against attacks from the host defence system [4,5]. It has also been observed that aminoglycoside antibiotics may trigger biofilm formation in some bacteria [6]. There is therefore an urgent need to design novel antibiotics against staphylococcus infections, especially in relation to biofilm development. Recently, the complete genome sequences of two *S. epidermidis *strains, viz. the non-biofilm-forming strain ATCC12228 and the biofilm-forming strain RP62A, have been published [7,8], bringing about new opportunities to discover potential antimicrobial targets using *in silico *genome analyses.

Two-component system (TCS) control proteins, harboring histidine kinase (HK) and response transcription regulator activities, have been uncovered in most bacteria. Recently, the TCSs have attracted attention due to their potential as novel antibacterial targets, especially those required for regulation of bacterial growth and virulence in pathogenic microorganisms [9,10]. One TCS, YycG/YycF, highly conserved and specific to low G+C Gram-positive bacteria has been shown to be essential for *Bacillus subtilis *and *Staphylococcus aureus *survival [11,12]. Inhibitors of the YycG HK, such as synthetic imidazole and zerumbone derivatives, or aranorosinol B, obtained by screening acetone extracts from 4000 microbes, have been documented to be effective antibacterial agents against *B. subtilis *[13,14]. Identification of this limited number of YycG inhibitors required laborious biological and chemical experiments, and the side-effects of these compounds on mammalian cells remain unclear. Moreover, *B. subtilis *may not be an optimal model organism to investigate biofilm formation, a process of major importance for the virulence of staphylococci. This prompted us to demonstrate that *S. epidermidis *possesses a homologous YycG/YycF TCS, and to investigate whether it would be an appropriate target for the design of novel antibacterial agents. As a prerequisite we set up a rapid and convenient procedure for screening novel inhibitors of the YycG/YycF TCS, testing the possible effects of these inhibitors on both planktonic and sessile bacteria, while using the extreme sensitivity of mammalian cells as a control to put aside compounds that would display a non-specific effect on membranes.

Upon binding, many small molecules may affect the functions of proteins. Functional analysis has been the basis of a variety of experiments, in which synthetic or purified small molecules have been used to probe the molecular mechanisms underlying the biological processes in which target proteins are involved. This chemistry-based approach has been coined "chemical biology" [15]. Combinatorial chemistry and *in vivo *or *in vitro *High Throughput Screening (HTS) constitute preferred approaches for discovering active compounds against particular protein targets [16]. A complementary approach is to use computational methods to identify active compounds (binders or hits) targeting the three-dimensional (3D) structure of the substrate binding pocket of a protein. This *in silico *approach is called Structure-Based Virtual Screening (SBVS) [16-19].

In the present study, we first identified the homologous YycG/YycF TCS in the genomes of the *S. epidermidis *ATCC12228 and RP62A strains. Next, a 3D structural model of the conserved HATPase_c domain of *S. epidermidis *YycG HK was constructed by using the homologous modeling approach. Subsequently, the SBVS method was used to search for potential YycG inhibitors from the SPECS chemical lead-compound database. Of the 76 candidates selected from the database by SBVS, seven compounds were active in inhibiting growth of *S. epidermidis *on plates or in liquid media. Five of these compounds displayed bactericidal effects on both planktonic and biofilm cells of *S. epidermidis*. Except for one, the compounds bound to the YycG protein and inhibited its auto-phosphorylation *in vitro*. These compounds displayed low cytotoxicity on mammalian cells and were not hemolytic, indicating that they may be good leads to develop new antibiotics against staphylococci infection.

## Results

### *In silico *identification of the YycG/YycF TCS in *S.epidermidis*

**Figure 1:**
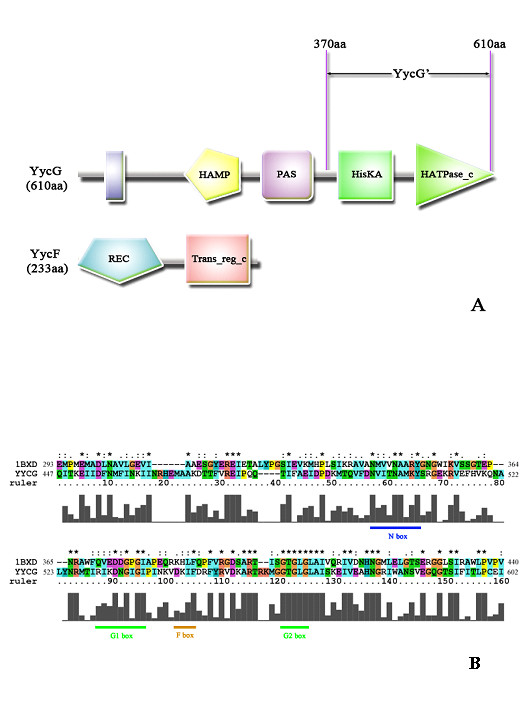
**Domain analysis of the YycG/YycF TCS of *S. epidermidis***. (A) Domain analysis of the two-component system (TCS) YycG/YycF of *S. epidermidis *ATCC12228. The analysis was performed based on the SMART database and the descriptions of putative functions of domains were also from SMART. HAMP: dimerization; PAS: FAD, heme, and cinnamic acid binding; HisKA: Phosphoacceptor, dimerization; HATPase_c: ATP-binding, Phosphorylation of HisKA domain, REC: phosphoacceptor, Trans_reg_C: DNA binding. The columns represent the transmembrane segment predicted by the TMHMM2 program, and the arrows indicate the start and end sites of the YycG protein fragment – YycG', as described in EXPERIMENTAL PROCEDUES. (B) The sequence alignment of the HATPase_c domain of YycG in *S. epidermidis *and that of EnvZ in *E. coli*. The height of columns below the alignment represents the similarity between two proteins. "*" denotes identical residues between two sequences, ":" means similar residues, "." means a bit different, and blank means completely different. Schematic alignment diagram was made by the program ClustalX.

Based on sequence homology, we identified likely counterparts of the YycG/YycF TCS (GenBank accession number: AY864800/AY864801) in the genomes of *S. epidermidis *ATCC12228 and RP62A. The sequence of the YycG protein in *S. epidermidis *is highly homologous to those of *S. aureus *and *B. subtilis *(92% and 46% identity, respectively). The same is true for the YycF protein (97% and 76% identity, respectively). As YycG and YycF are "persistent" proteins in Firmicutes [20] we can be confident that their function is preserved in the clade. More importantly, attempts to inactivate the orthologous *yycG*/*yycF *genes identified in *S. epidermidis *(this work) also failed with homologous recombination technology, indicating that this TCS is required for bacterial growth of *S. epidermidis *(data not shown). Domain analysis indicates that the YycG protein of *S. epidermidis *contains one transmembrane segment, and several domains identified in other proteins: HAMP (a domain present in Histidine kinases, Adenylyl cyclases, Methyl-accepting proteins and Phosphatases), PAS (a domain initially found to be common to Period circadian protein, Ah receptor nuclear translocator protein, and Single-minded protein), HisKA, and HATPase_c, all common to bacterial HKs [21]. Moreover, the YycF protein of *S. epidermidis *possesses a conserved REC domain containing a phosphoacceptor site that may be phosphorylated by YycG, and a Trans_reg_C domain, belonging to the OmpR-like winged helix-turn-helix DNA-binding domains (Figure 1A). Interestingly, multi-alignment of HATPase_c domain sequences with counterparts of Bacteria from the structural Protein Data Bank (PDB) showed that the sequences around the ATP binding site in most bacteria were similar, showing four conserved important motifs – the N box, G1 box, F box and G2 box [22]. This demonstrated that the ATP binding domain of HKs is highly conserved in bacteria, suggesting that it may be used as a potential target for antibacterial agents screening [23]. Comparison of the YycG HATPase_c domain of *S. epidermidis *with similar domains in the database showed that the most homologous sequence was the similar domain of the *E. coli *osmosensor EnvZ, a TCS molecule, with 30% sequence identity and 49% conservative replacements (Figure 1B). EnvZ was therefore used as a template for modeling the 3D structure of the YycG HATPase_c domain of *S. epidermidis*.

### A 3D model of the YycG HATPase_c domain of S. epidermidis

**Figure 2:**
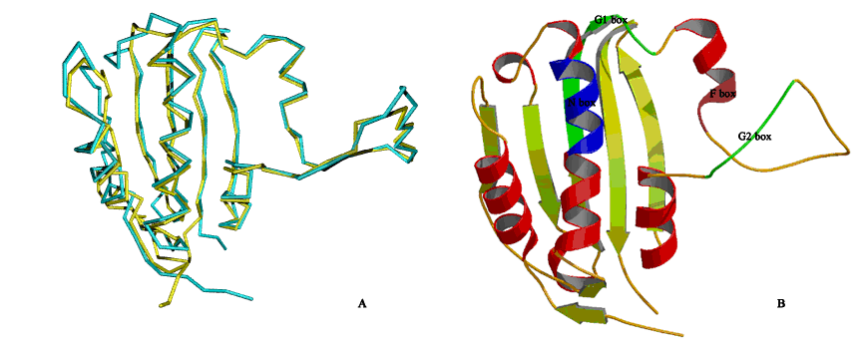
**The modeled structure of the YycG HATPase_c domain of *S. epidermidis***. (A) Structure superposition of the modeled structure of YycG HATPase_c domain of *S. epidermidis *(blue) with the NMR structure of the homologous domain of EnvZ in *E. coli *(yellow). Only backbones are shown in this picture. (B) The solid ribbon representation of the structure model of the YycG HATPase_c domain. The YycG HATPase_c domain of *S. epidermidis *folds into a two-layer sandwich structure. Four high conserved motifs, N-box (Asn499~Tyr507), G1-box (Ile53~Ile538), F-box (Asp544~Phe547), G2-box (Gly563~Gly567), around the catalytic domain of the HPK encompasses the active ATP-binding pocket and a long loop from Asp548 to Ala574 drifts outside the pocket. The substrate-binding site is located at the deep cleft among N, G1, F, G2 boxes. Schematic diagrams were made by the program Molscript.

**Figure 3:**
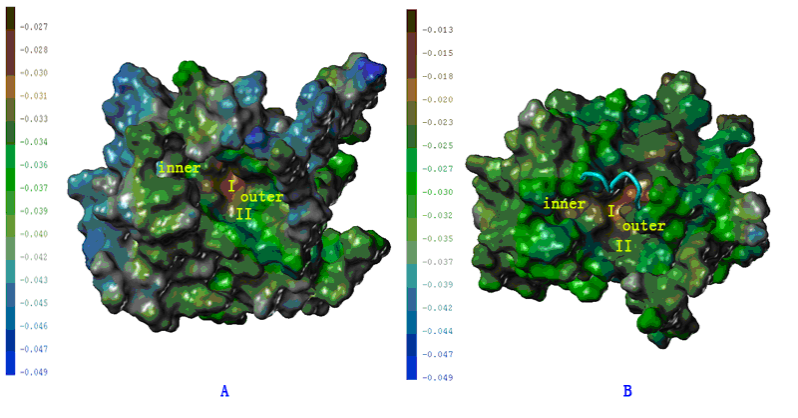
**Shape and surface features of the ATP-binding pocket of the YycG HATPase_c domain in *S. epidermidis***. (A). View from the front of the pocket of the HATPase_c domain. (B) View from the top of the pocket of the HATPase_c domain. To display the bottom of the pocket clearly, some residues which cover the top of the pocket were taken off from the surface. The ATP binding pocket is fairly large and deep. Two cavities joined by a gorge-like channel construct the whole binding pocket. The inner small cavity of the pocket is hydrophobic, composed of residues Phe498, Val501, Phe56, Ile53, and the adenine ring of natural ligand ATP may interact with this area just as in the NMR structure of EnvZ[24]. The space compressed by Asn503 and Lys542 in the middle of the pocket form the narrow channel. The outer large cavity of pocket divides into two parts (I and II) in terms of its surface property. Area I composed of residues Phe46, Thr483, Ile484, Phe485, Met493, and Leu598, exhibits hydrophobic character; Area II locates near entrance of the binding pocket and show hydrophilic character. Schematic diagrams were made with the MOLCAD program in Sybyl (see Methods).

To search for potential inhibitors of YycG HK by virtual screening, we constructed a 3D model for the YycG HATPase_c domain of *S. epidermidis *based on the NMR structure of the homologous domain of the *E. coli *osmosensor EnvZ (PDB entry 1BXD) [24]. The resulting model 3D structure is shown in Figure 2. The final structure was checked and validated using several programs such as Prostat and Profile-3D [25]. This model superposed well with the NMR structure of the homologous domain of EnvZ, the root-mean-square deviation (RMSD) for the Cα atoms being about 1.708 (Figure 2A). The HATPase_c domain of YycG is thus predicted to fold in a similar way to that in EnvZ, containing five stranded β-sheets and four α-helices, which form a two-layered α/β sandwich structure (Figure 2B). The surface shape and the general features of the HATPase_c domain of YycG were further investigated by using the MOLCAD module of Sybyl 6.8 [26]. The ATP binding site consists of two different cavities connected by a gorge-like channel (Figure 3). This structural prediction was used for virtual drug screening, bearing in mind that only further experimental evidence would validate the model.

### Discovery of potential inhibitors of the *S. epidermidis *YycG HK by Virtual Screening

The ATP-binding pocket formed by residues within a radius of 5 Å around the ATP site of the YycG HATPase_c model of *S. epidermidis *was used as the target site for high throughput virtual screening (HTVS). In a first step, 85,000 potential drug-like molecules, constituting an in-house database (named SPECS_1), were selected from the SPECS database using the drug-selection filter developed by Zheng *et al*. [27]. The SPECS_1 database was searched for potential binding molecule structures using the program DOCK4.0 [28,29] in a primary screening. The most optimal 10,000 structures were subsequently re-scored using the FlexX program [30] and CSCORE [31], a consensus scoring method that integrates five popular scoring functions. Two hundred molecules passed this highly selective filter. Finally, 100 molecules were manually selected from the latter sample as inhibitor candidates, according to their molecular diversity, their shape complementarity, and their potential for forming hydrogen bonds in the binding pocket of the YycG HATPase_c domain. Of those 100 candidates, 76 compound samples could be purchased from the SPECS Company for further experimental assays.

### Antimicrobial activities of potential YycG inhibitors *in vitro*

**Figure 4:**
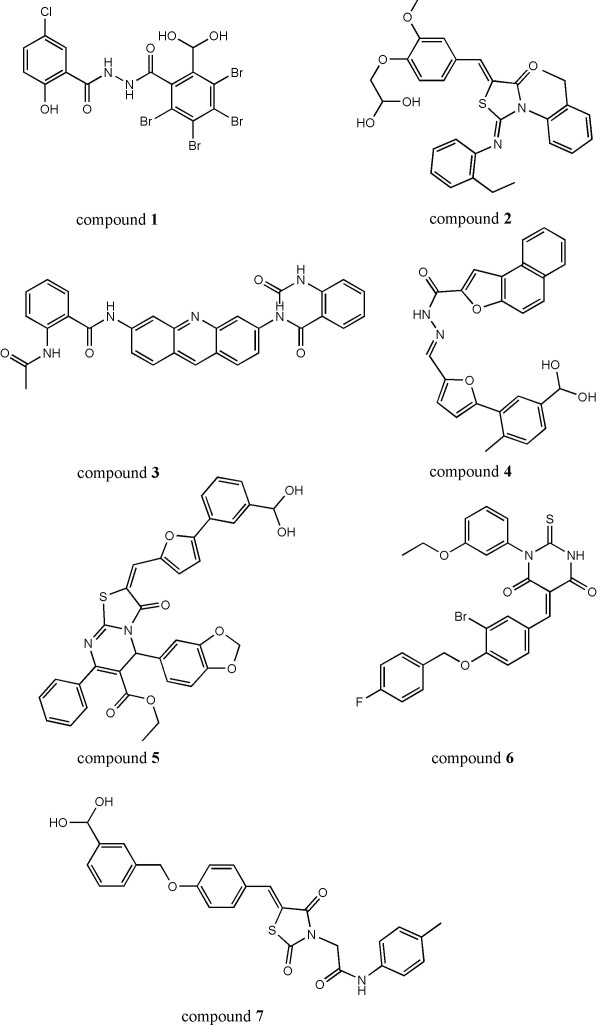
**The chemical structures of seven antibacterial compounds as potential YycG inhibitors**. These compounds include three derivatives of thiazolidinone (compounds **2**, **5**, and **7**), two derivatives of benzamide (compounds **1 **and **3**), one derivative of furan (compound **4**) and one derivative of pyrimidinone (compound **6**).

Since the YycG/YycF TCS is essential for growth and survival in *B. subtilis *and *S. aureus *[11,12], its conservation in sequence and in genome organization in *S. epidermidis *strongly suggests that it is essential in this organism as well. To test this possibility, we explored whether the potential YycG inhibitor candidates obtained by virtual screening could inhibit bacterial growth. At a concentration of 200 μM in liquid culture in a first screening procedure, seven out of the 76 candidates completely inhibited growth of *S. epidermidis*. The seven inhibitors belong to four different classes of chemical structures: three thiazolidinone analogs (compounds **2**, **5**, and **7**), two benzamide analogs (compounds **1 **and **3**), one furan derivative (compound **4**) and one derivative of pyrimidinone (compound **6**), as shown in Figure 4.

Subsequently, the Minimal Inhibitory Concentration (MIC) values of these 7 compounds were determined, using the standard tube-dilution assay (Table 1). These 7 compounds were further shown to have similar MIC values when tested against 4 clinical isolates of *S. epidermidis *from different patients in the Zhongshan Hospital of Shanghai, China (data not shown). Compounds **1**–**6 **showed similar MIC values (see Table 1) on the *S. epidermidis *non-biofilm-forming strain ATCC12228 and on the biofilm-forming strain RP62A, whereas compound **7 **was only effective against the non-biofilm-forming strain ATCC12228. We also investigated their inhibitory effect against other Gram-positive pathogenic cocci which possess the homologous YycG/YycF TCS. This experiment showed that compounds **2 **and **5 **were effective against *S. aureus*, *Streptococcus pyogenes *and *Streptococcus mutans*, compound **1 **and **3 **inhibited the growth of *S. aureus *and *S. pyogenes*, and compound **4 **was only active against *S. pyogenes*. In contrast, none of these compounds were active against Gram-negative bacteria, such as *Escherichia coli *or *Pseudomonas aeruginosa *at the concentration of 200 μM. This is consistent with the absence of a YycG/YycF TCS counterpart in these genomes and, taken together, this is a first observation suggesting that these compounds are not likely to act by trivial interaction with membrane structures. Subsequently, we investigated the bactericidal activity of these compounds against *S. epidermidis *using a standard Minimal Bactericidal Concentration (MBC) assay. Five compounds (**1–5**) had their MBC values (about 4 times MIC) below the threshold (200 μM), and no obvious differences were observed between the biofilm-forming strain and non-biofilm-forming strain of *S. epidermidis *(Table 1).

### Killing biofilm cells of *S. epidermidis *by potential YycG inhibitors

**Figure 5:**
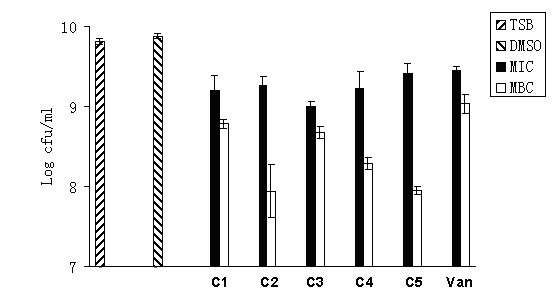
**Killing biofilm cells of *S. epidermidis *by potential YycG inhibitors**. Mature biofilm of *S. epidermidis *RP62A strain were formed in 12-wells polystyrene plates (see Methods). The effect of various compounds (dissolved in DMSO) on biofilm-covered cells was investigated: TSB medium (control, the first column); TSB medium with an equal volume of DMSO solution (control, the second column); TSB medium with various compounds at their MIC values (black columns); TSB medium with various compounds at their MBC values (white columns). This assay was repeated three times and the values represented the mean and SD from three experiments. The concentrations we used in this assay (MBC/MIC): C1 (200 μM/50 μM), C2 (100 μM/25 μM), C3 (100 μM/25 μM), C4 (100 μM/12.5 μM), C5 (25 μM/6.25 μM), and Van (4 μg/ml/1 μg/ml), according to the results from MBC/MIC assays with planktonic cells of *S. epidermidis *RP62A strain. C: compound, Van: vancomycin.

Standard MIC/MBC assays measure anti-microbial activities on planktonic bacteria, whereas antibiotic resistance is reported to be enhanced up to 1000-fold in the same cells when they develop in biofilms [32]. We therefore measured the bactericidal effect of compound (**1–7**) on sessile cells of *S. epidermidis*. Vancomycin, one of the antibiotics usually used in multidrug-resistant staphylococci associated infections [33], was as a comparison (MBC/MIC = 4 μg/ml/1 μg/ml in *S. epidermidis *RP62A strain). Compared with vancomycin, the potential YycG inhibitors (compounds **1–5**) showed better killing efficiencies against 24-hour-old biofilm cells of *S. epidermidis *at their MBC values, respectively (Figure 5). The best chemical (compound **5**) reduced the CFU of the biofilm cells more than 100-fold (relative to an untreated control). In contrast, vancomycin caused no increased killing of the biofilm cells even when the concentration was increased to 128 μg/ml, relative to that of its MBC value of 4 μg/ml (data not shown). In addition, recent data by using confocal laser scanning microscope (CLSM) also confirmed that these compounds exhibited much better killing effects than vancomycin on cells embedded in mature biofilms of *S. epidermidis *clinical isolates (data not shown.

### Binding affinity of potential YycG inhibitors to the YycG' protein

**Figure 6:**
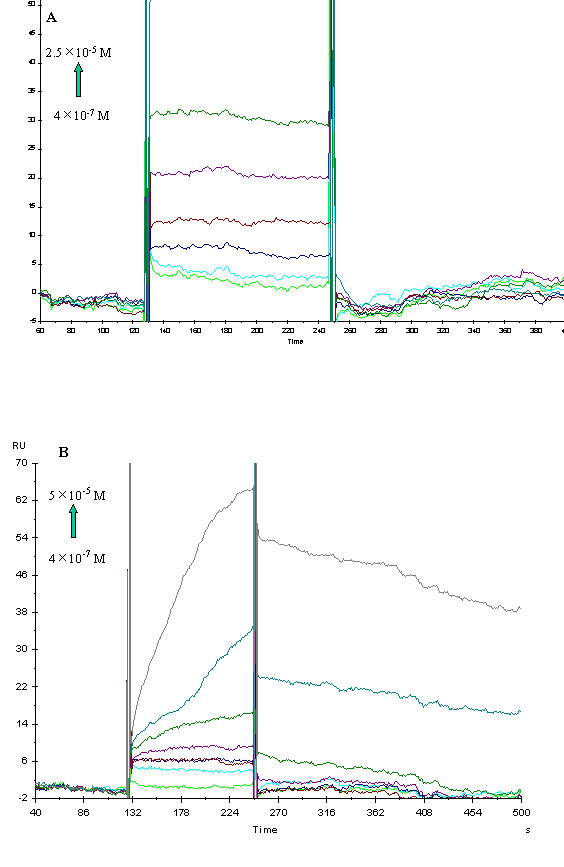
**Binding affinities of the potential YycG inhibitors to the YycG' protein determined by using SPR**. Real-time measurement of the interactions of compounds **1 **(A) and **7 **(B) to the YycG' protein was done by using the Biacore 3000 instrument. The curves represented the interaction of various concentrations of compounds (shown in the figures) with the protein. The compounds were injected for 120 s, and dissociation was monitored for more than 150s (see Methods).

The *in vivo *effects of the inhibitors extracted from the *in silico *screening procedure are consistent with YycG being their target. However, there is no straightforward extrapolation from *in silico *design to accurate identification of a target *in vivo*. In order to confirm the interaction of the potential YycG inhibitors with their putative target protein, recombinant His-tagged YycG' protein (the fragment of YycG protein used in the *in silico *approach, approximately 34 kDa as indicated in Methods) was overproduced by using the pET28a plasmid, and purified using the ProBond™ Purification System (see Additional File 1, the protein purity is above 95%). The binding affinities of the seven putative YycG inhibitors to the YycG' protein were determined by using Surface Plasmon Resonance (SPR) *in vitro*. Six compounds (**1**–**5 **and **7**) displayed high binding affinities to the YycG' protein (Table 2), whereas compound **6 **only bound very poorly to the YycG' protein. All the 6 positive compounds displayed a significant and dose-dependent binding pattern in the SPR response. Five compounds (**1**–**5**) showed characteristic square-wave binding curves, indicating that these compounds form unstable complexes with the YycG' protein in a process of fast association and fast dissociation (only compound **1 **was shown in Figure 6A). Compound **7 **displayed a slow association and slow dissociation reaction, forming a relatively stable complex with the YycG' protein (Figure 6B). The data were fitted to a steady-state affinity model for compounds **1**–**5 **and a kinetic-state model for compound **7 **using the Biacore 3000 software to evaluate the corresponding binding affinities (*K*_D _values), as shown in Table 2. The *K*_D _value of compound **6 **can not be calculated because of its very poor binding affinity to the YycG' protein.

### Inhibition of the YycG' protein ATPase activity *in vitro*

The common characteristic of HKs is ATP-dependent auto-phosphorylation, associated with the conserved HATPase_c domain. We measured the effects of our potential YycG inhibitors on the protein ATPase activity by using the Kinase-Glo™ Luminescent Kinase assay. Firstly, the putative kinase activity of YycG' protein was measured by quantifying the amount of ATP remained in solution after reaction. A direct relationship existed between the luminescence measured with the Kinase-Glo™ Reagent and the amount of ATP (see Additional File 2A), indicating the sensitivity of this assay is good. After adding purified YycG' protein into the reaction system (4 μg protein in each reaction system), the luminescence was decreased in all the groups containing different ATP concentrations, compared with parallel groups without YycG' treatment (see Additional File 2B), since the kinase can hydrolyze ATP for its auto-phosphorylation. Keeping the ATP concentration constant (50 μM), the luminescence was continuously decreased following the amounts of purified YycG' protein increased (see Additional File 2C). The results indicated that the purified YycG' protein possesses the ATPase activity *in vitro*. At a concentration of 50 μM, compounds **1–5 **and **7 **decreased the ATPase activity of 4 μg YycG' protein in the presence of 3 μM ATP by 52% to 86% (52%, 61%, 70%, 76%, 73% and 86%, respectively), indicating that the binding affinities of these compounds to YycG' correlate well with their inhibitory activities of auto-phosphorylation of YycG'. In contrast, compound **6, **which binds poorly to the YycG' protein, showed almost no activity against YycG' ATPase activity (approximately 7% inhibition at 50 μM). The concentrations needed to decrease YycG' ATPase activity to 50% (IC_50 _values) by the 6 active compounds (**1–5 **and **7) **were calculated by gradual dilution of these compounds with an invariable concentration of protein (4 μg) and ATP (3 μM), as shown in Table 2. The IC_50 _value of compound **6 **was above 200 μM under the same reaction conditions. As a comparison, the fragment of another HK protein SrrB' containing homologous domains with YycG' in *S. epidermidis *[45] was also expressed and purified to observed the inhibitory effect on protein phosphotylation by these 6 potential YycG inhibitors (**1–5 **and **7) **(Supple. Table 1). At the concentration of 50 μM, only compound **5 **displays much lower inhibitory effect (20%) on SrrB' than it does (73%) on YycG', while the other 5 compounds have almost no inhibitory effects.

### Cytotoxicity and hemolysis of the antimicrobial compounds *in vitro*

**Figure 7:**
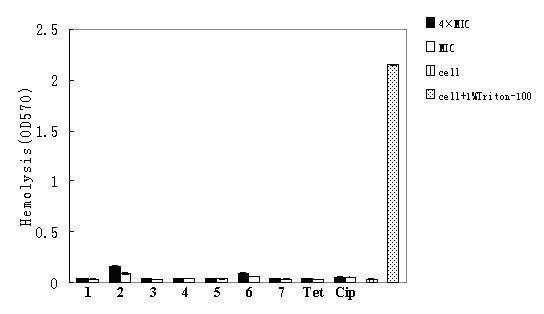
**Hemolytic activities on healthy human erythrocytes of 7 potential YycG inhibitors**. The MICs of 7 inhibitors are compound **1 **(50 μM), compound **2 **(25 μM), compound **3 **(12.5 μM), compound **4 **(12.5 μM), compound **5 **(6.25 μM), compound **6 **(100 μM), and compound **7 **(0.2 μM), respectively. The MICs of Tet and Cip are both 0.25 μg/ml. Each assay was performed in quadruplicate and repeated twice. The values represented the mean and SD from one separate experiment. Cells with no compounds treatment and with 1% Triton-100 treatment were for the zero and 100% hemolysis controls, respectively. Black and white columns represented the concentrations of 4×MICs and MICs of 7 inhibitors and two conventional antibiotics, respectively. Tet: tetracycline, Cip: ciprofloxacin.

While several of the compounds we identified could be used as excellent drug leads, acting on targets that are absent from eukaryotic cells, they still may display toxicity by interfering with unexpected targets. As a step to rule out this possibility we analyzed the cytotoxicity of compounds **1–7 **on a Vero cell line (Vero 76, African Green-monkey) by using the traditional 3-(4,5-dimethylthiazol-2-yl)-2,5-diphenyl tetrazolium bromide (MTT) method (the Cell Proliferation Kit I, Roche, see Methods). This approach would also provide us a very sensitive assay to draw aside a trivial action of these products on membrane lipid bilayers. Remarkably, as shown in Table 2, most CC_50 _values were higher than the highest inhibitor concentration used (200 μM), except in the case of two inhibitors (compounds **2 **and **3**) which had CC_50 _values below 200 μM (CC_50 _is the concentration that induces a 50% cytotoxicity effect on Vero cells). Furthermore, at the concentration of their respective MIC values, all these compounds displayed very low (< 10%) or almost no cytotoxicity (data not shown). It has been reported that some TCS inhibitors induce hemolysis of mammalian erythrocytes, which has hindered the further development and application of these inhibitors [34]. This prompted us to analyze the possible membrane damage caused by our potential YycG inhibitors by examining hemolysis of healthy human erythrocytes possibly induced by these compounds. At their MIC concentrations, compounds **1–7 **displayed no apparent hemolysis (< 5% of the zero control). This compares well with the effect of conventional antibiotics such as tetracycline and ciprofloxacin (Table 2 and Figure 7). At their 4 times MIC concentrations, only compound **2 **showed a little hemolysis (~6% of the zero control), and at the highest concentration (200 μM), the hemolytic activity of compound **2 **was increased to ~16% of the zero control.

### Interaction models of potential YycG inhibitors to the target protein

As potential YycG inhibitors, six compounds (**1**–**5 **and **7**) selected by *in silico *screening bound with high affinity to the YycG' protein and inhibited its ATPase activity *in vitro*. To further investigate the sites of interaction between the compounds and the YycG protein, and to develop a strategy for designing novel inhibitors, models of the interaction of the compounds with the YycG protein were analyzed based on docking simulations.

**Figure 8:**
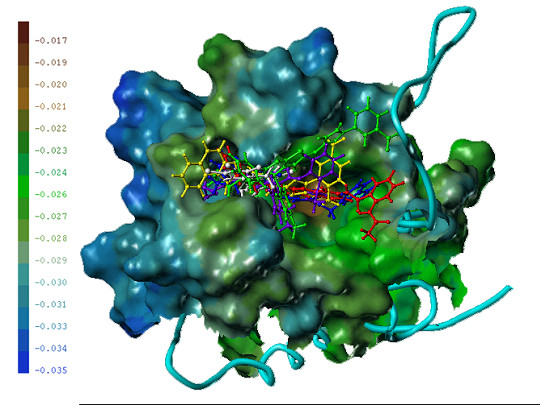
**Interaction models of the six potential YycG inhibitors to the HATPase_c domain of YycG protein**. Six potential YycG inhibitors (compounds **1**–**5 **and **7**) were docked into the ATP-binding pocket of YycG protein. Surface of the pocket was made by the MOLCAD program in Sybyl. They interact with residues in the binding site by a rather similar mode. Inhibitors were figured using stick mode with different colors: compound **1 **(white), compound **2 **(purple), compound **3 **(red), compound **4 **(yellow), compound **5 **(green), and compound **7 **(blue).

**Figure 9:**
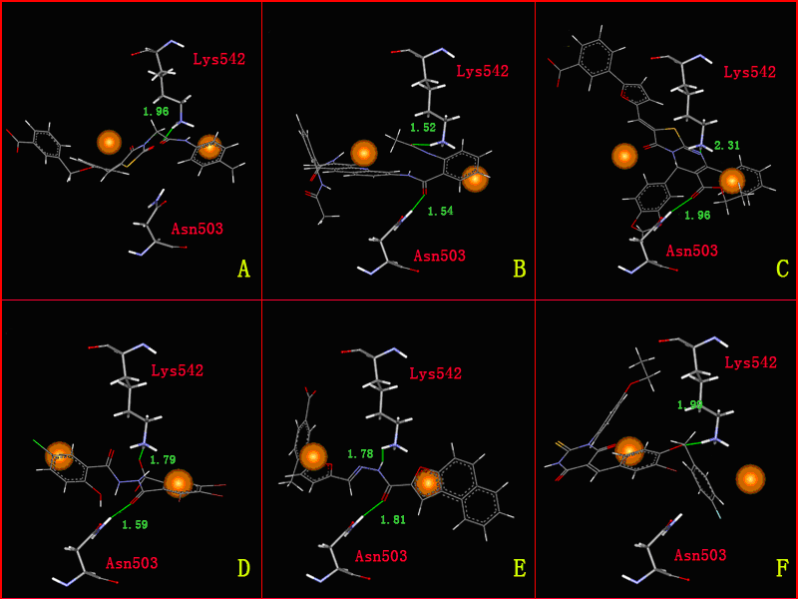
**Four-point pharmacophores model for the six potential inhibitors binding to the YycG protein**. Four-point pharmacophores models of inhibitors compose of two hydrogen bond acceptors, which interact with Asn503 and Lys542, and two hydrophobic centers (orange spheres) on the both side of gorge of the binding site, which interact with hydrophobic residues, it was constructed by DISCO in Sybyl based on the six inhibitors and could provide useful information for novel inhibitor design and structural modification. A (compound **1**); B (compound **2**); C (compound **3**); D (compound **4**); E (compound **5**); F (compound **7**).

The surface of the YycG HATPase_c domain shows two significant hydrophobic areas located in the ATP-binding pocket. One is placed in the inner small cavity and is composed of residues Phe498, Val501, Phe56 and Ile53; the other is positioned in the front of the outer larger cavity, consisting of residues Phe46, Thr483, Ile484, Phe485, Met493, and Leu598 (Figure 3). Most of these residues are conserved in bacterial HKs. The binding conformations of the inhibitors designed in the present study in the ATP-binding pocket of the YycG HATPase_c domain are shown in Figure 8. Although their structures are diverse, they adopt similar interactions with the conserved domain. According to the interaction models, a four-point pharmacophore can be figured out for describing the binding of the inhibitors (Figure 9): the middle part of each inhibitor has two hydrogen bond acceptors, hydrogen bonding to Asn503 and Lys542, which stabilize each one of the six inhibitor candidates in the binding pocket; the hydrophobic moieties of the inhibitors fit well into the two hydrophobic cavities. Noticeably, the binding model revealed that Asn503 is of prime importance for stabilizing the interactions between the inhibitors and the YycG protein. This is well in agreement with previous biological function studies which demonstrated that the conserved Asn503 in the N box was necessary for ligand binding in both classes I and II TCS [35,36]. In contrast, Lys542 is a residue specific to YycG. Our binding models have provided new possibilities for studies of the biological function of Lys542 in ligand binding and catalysis of YycG (Figure 9). Besides those interactions, the hydrophobic area (mainly constructed by residues Val500, Ile536, Ile538, Pro539, Thr592 and Ile594) may bind hydrophobic parts of different compounds and stabilize these in the inner hollow of the pocket. Most of these residues are conserved in homologous HKs (data not shown).

## Discussion

The YycG/YycF TCS is conserved and specific for low G+C Gram-positive bacteria such as *B. subtilis*, *S. aureus*, *Streptococcus pneumoniae *and *Listeria monocytogenes *[11,12,37,38]. This TCS has been shown to be essential in *B. subtilis *[39] and could not be inactivated by direct mutation in *S. aureus *[12], and which also occurs in *S. epidermidis *(this work). Moreover, several genes involved in cell-wall biosynthesis and metabolism, such as teichoic acid biosynthesis protein F [40], and cell wall synthesis protein YpfP [41] were predicted to be regulated by this TCS in *S. epidermidis *based on the presence of a potential YycF consensus DNA binding recognition sequence similar to the one described in *B. subtilis *and *S. aureus *[42,43], although this predicted result needs to be further verified (Qin *et al*, unpublished data). We have therefore explored the possibility of using the YycG HK as a potential target in a screening for new antibiotics.

Compared with biological and chemical screening for new antibiotics, the advantage of SBVS technology is the rapid, economical and efficient throughput. Furthermore, lead-compound databases provide ample sources for screening, whereas chemical synthesis is time consuming and expensive. As a case in point, the use of lead-compound databases has already led to the discovery of the diarylquinoline lead-compound against *Mycobacterium tuberculosis *[44].

Screening 80,000 possible compounds *in silico *we finally retained 7, among which 6 were promising candidates as potential YycG HK inhibitors. These 6 compounds bound to the YycG protein *in vitro *and inhibited its ATPase activity, while they were also active antimicrobials against *S. epidermidis*. This does not prove, however, that YycG is the only target when compounds interact with bacteria. In fact, we have found a total of 16 putative TCSs in the genome of *S. epidermidis*, including YycG/YycF. Because they have many core features in common, we cannot exclude that some of the compounds may also bind to other HKs. This would in fact enhance the antibiotic effect of the compounds. To investigate this possibility, we expressed and purified the fragment of another HK of *S. epidermidis *– SrrB (designated as SrrB' containing similar domains with YycG' described in Methods), which is involved in the TCS SrrB/SrrA [45]. We analyzed the effects of the 6 active compounds (**1–5 **and **7**) on the auto-phosphorylation of this purified protein. We observed that only compound **5 **(at 50 μM) inhibited the auto-phosphorylation of SrrB'. The inhibition was approximately 20% lower than that demonstrated here on YycG' (73%) under the same reaction conditions (see Additional File 3). This suggests that the compounds display a certain degree of specificity dependent on the characteristics of the HATPase_c domain structure in the different HKs.

One of the interesting and challenging goals of new searches for anti-microbial compounds is to identify potential drugs which are equally active against planktonic and sessile bacteria (biofilms). The YycG inhibitor compounds described here were found to have somewhat reduced bactericidal effect on mature biofilms of *S. epidermidis*. These compounds were, however, much more efficient against sessile bacteria than the commonly used staphylococcus antibiotic, vancomycin. In fact, vancomycin was almost without effect against biofilm cells (even at 128 μg/ml), as also reported by others [46]. This was accounted for in *S. aureus *by reduced penetration of vancomycin and delay of the exposure of the bacteria in the biofilm deeper layers [47]. However, it remains unknown whether the situation is the same with our compounds in *S. epidermidis *biofilms. Interestingly, seventeen compounds among the 76 candidates we retained for further studies were active in inhibiting biofilm formation without interfering with bacterial growth. Most of them did not bind to the YycG' protein nor inhibit its auto-phosphorylation *in vitro*, indicating that these compounds are not potential inhibitors of the YycG HK (data not shown). Their targets in *S. epidermidis *and the mechanisms of inhibiting biofilm formation are still under investigation.

In previous studies, some potential inhibitors of histidine kinase appeared to display trivial side effects, such as membrane disruption, excessive protein binding or limited bioavailability. This prevented their further development [34]. In this study, the potential YycG inhibitors displayed low cytotoxicity and low hemolysis to mammalian cells at the effective concentrations we used *in vitro*. We are now in the process of finding out the more effective derivatives, using those as leads. In future work, an appropriate animal model also needs to be established to investigate the effect of these compounds *in vivo*.

## Conclusion

In our study, these novel inhibitors of YycG histidine kinase are considered as promising lead-compounds for developing new reagents against staphylococci infections. Furthermore, the structure-based virtual screening (SBVS) technology can be widely used for discovery of potential bacterial TCSs inhibitors in both Gram-positive and Gram-negative species, even on other "drug target" proteins. And it is more rapid and efficacious than traditional screening technology.

### The abbreviations used are

TCS, two-component system; HK, histidine kinase; SBVS, structure-based virtual screening; HTS, high throughput screening; MIC, minimal inhibitory concentration; 3D, three-dimensional; SPR, surface plasmon resonance.

## Methods

### Bacterial strains, media and reagents

*Staphylococcus epidermidis *ATCC12228 and RP62A strains were purchased from the American Type Culture Collection (ATCC, Manassas, USA). *Staphylococcus aureus *ATCC29213, *Escherichia coli *ATCC25922 and *Pseudomonas aeruginosa *ATCC27853 were kindly provided by Dr. Bijie Hu at Zhongshan Hospital (Shanghai, China). *S. epidermidis *strains se527, se886, se847, seG203, *Streptococcus pyogenes *strain 144, and *Streptococcus mutans *strain 128 were all clinical isolates from Zhongshan Hospital and Huashan Hospital (Shanghai, China). If not stated otherwise, *S. epidermidis *and *S. aureus *strains were grown at 37°C in tryptic soy broth (TSB, Oxoid) containing 0.25% glucose, *E. coli *and *P. aeruginosa *strains were grown at 37°C in Luria-Bertani (LB, Oxoid).

All compounds used as inhibitor candidates were purchased from the SPECS Company in the Netherlands. Stock solutions of the compounds were prepared in dimethyl sulfoxide (DMSO). All other chemicals were of reagent grade or ultra-pure quality and purchased from Sigma.

### Bioinformatics analysis

Domain analysis was performed based on the SMART database [48]. The complete genome sequences of the two *S. epidermidis *strains, ATCC12228 (NC_004461) and RP62A (NC_002976) were accessed from the National Center for Biotechnology Information (NCBI) genome database [49]. The homologous sequences with the YycG HATPase_c domain of *S. epidermidis *were searched from the Protein Data Bank (PDB) [50], using the BLASTp program [51]. ClustalX was used to align the protein sequences [52].

### 3D structure modeling of the YycG HATPase_c domain

The sequence of *S. epidermidis *HK YycG was retrieved from GenBank (accession number AY864800). The Align123 module encoded in InsightII [53] was used in the pair-wise sequence alignment. Using the secondary structure information of EnvZ (PDB entry 1BXD) [24], the sequence alignment was adjusted manually to obtain a fine alignment for 3D structure construction. The 3D model of the YycG HATPase_c domain was generated by using the MODELLER program [54] encoded in InsightII. Finally, the whole structural models were optimized using the Discover_3 module of InsightII with CVFF force field. Several structural analysis softwares such as Prostat and Profile-3D [25] were used to check the structure quality. The Prostat module of InsightII was used to analyze the properties of bonds, angles, and torsions. The Profile-3D program was used to check the structure and sequence compatibility.

### Structure-based virtual screening

Before docking the small molecules of interest on the model structure, we delineated the general features that the binding pocket should have. Major residues possibly composing the ATP-binding site of YycG HATPase_c domain were identified by the sequence alignment with the osmolarity sensor protein EnvZ of *E. coli*, and the ATP-binding pocket was probed on the optimized 3D model of YycG HATPase_c domain using the SiteID program encoded in Sybyl6.8 [26]. The surface (electrostatic, hydrophobic and hydrogen bonding) properties of the binding pocket of the YycG HATPase_c domain were calculated using the MOLCAD program encoded in Sybyl6.8. The ATP-binding pocket of the YycG HATPase_c domain was used as a target for screening the SPECS database using the docking approach [55]. The SPECS database contains the structural information of 280,000 small molecules. The SPECS Company supplies all the compound samples collected from different sources. As a first step, the SPECS database was submitted to our own filter of drug-ability [27], non-drug-able molecules were eliminated from the database, and finally 85,000 potential drug-able molecules were selected out for docking screening. The program DOCK 4.0 [28,29] was used for primary screening. Residues within a radius of 5 Å around the ATP-binding pocket of the YycG HATPase_c domain were used for constructing the grids for the docking screening. During the docking calculations, Kollman-all-atom charges [56] were assigned to the protein, and Gasterger-Hückel charges [57,58] were assigned to the small molecules. Conformational flexibility of the compounds from the database was implemented in the docking search. During DOCK simulation, the ligand-receptor binding energy was approximated by the sum of the van der Waals and electrostatic interaction energies. After an initial evaluation of orientation and scoring, a grid-based minimization was carried out for the ligand to locate the nearest local energy minimum within the receptor binding site. Position and conformation of each docked molecule were optimized using the single anchor search and torsion minimization method of DOCK 4.0. The 10,000 compounds with the highest score as obtained by DOCK search were selected for a second round docking using the FlexX program [30], and CSCORE [31] was used to re-score the compounds. The virtual screening was performed on a 392-processor Sunway-1 supercomputer at the Shanghai Supercomputer Center.

### Minimal inhibitory concentration (MIC) and minimal bactericidal concentration (MBC) assays

MIC assays for the antibacterial activities of the compounds were performed according to the broth micro-dilution (in tubes) method of the Clinical and Laboratory Standards Institute (CLSI) of America [59]. The Minimal Bactericidal Concentration (MBC) was obtained by subculturing 100 μl from each negative (no visible bacterial growth) tube from the MIC assay, onto substance-free Mueller-Hinton agar plates. The plates were incubated at 37°C for 24 hours, and the MBC was defined as the lowest concentration of substance which produced subcultures growing no more than five colonies on each plate.

### Killing biofilm cells of S. epidermidis by potential YycG inhibitors

An overnight culture of *S. epidermidis *strain RP62A was diluted 1:100 in TSB containing 0.25% glucose, then 1 ml bacterial suspension was inoculated into the wells of sterile 12-well polystyrene microtiter plates (Falcon) incubated at 37°C for 24 h. The plates with mature biofilm were washed gently four times with sterile PBS before adding fresh TSB containing the various compounds at their MBC values, and incubated at 37°C for 24 h. The plates were washed again four times with sterile PBS, then the biofilm cells were scraped from the plate and resuspended in 1 ml PBS, and violently vortexed to disintegrate clumps of cells. Next, the suspension was diluted gradually with sterile PBS and subcultured onto substance-free Mueller-Hinton agar plates, incubated at 37°C for 24 h, and the colonies were counted. The fresh medium containing vancomycin at the MBC value and substance-free fresh medium served as the controls. The experiment was repeated three times.

### Cloning, expression and purification of the YycG' protein

The YycG' fragment containing the cytoplasmic signal domains (the HATPase_c and HisKA domain, see Figure 1A) of YycG (370aa to 610aa) was amplified by PCR (with the chromosomal DNA of *S. epidermidis *ATCC12228 as the template, and (5' GCGGATCCACAACAACAAGTCGAACGTGAAC 3') and (5' GCCTCGAGTTATTCATCCCAATCACCGTCT 3') as the primers. Subsequently, the fragment was digested with BamHI and XholI (TAKARA, Japan) and ligated into the corresponding sites of pET28a (Promega, Madison, USA) to obtain pETYycG'. The expressed YycG' protein was purified with the ProBond™ Purification System (Invitrogen, California, USA), according to the manufacturer's protocol.

### Compound-YycG' protein binding assay

The in vitro binding affinities of the inhibitor compounds to the YycG' protein was determined using surface plasmon resonance (SPR) biosensor technology on the dual flow cell Biacore 3000 instrument (Biacore AB, Uppsala, Sweden) with a similar method as described in our previous study [60]. Immobilization of the YycG' protein to the hydrophilic carboxymethylated dextran matrix of the sensor chip CM5 (Biacore) was carried out by the standard primary amine coupling reaction. The protein to be covalently bound to the matrix was diluted in 10 mM sodium acetate buffer (pH 4.2) to a final concentration of 0.3 mg/ml. Equilibration of the baseline was completed by a continuous flow of HBS-EP running buffer (10 mM HEPES, 150 mM NaCl, 3.4 mM EDTA and 0.005% (v/v) surfactant P20, pH 7.4) through the chip for 1–2 hours. Biacore data were collected at 25°C with HBS-EP as running buffer at a constant flow of 20 ml/min. Sensorgrams were processed by using automatic corrections for non-specific bulk refractive index effects. The equilibrium constants (*K*_D_) evaluating the protein-ligand binding affinity were determined by the steady state or the kinetic state affinity fitting model encoded in the Biacore analysis software.

### Inhibition assay for the ATPase activity

The inhibitory activities of the compounds for the ATPase activity of the YycG' protein was measured using the Kinase-Glo™ Luminescent Kinase Assay (Promega, Madison, USA). Briefly, 4 μg YycG' protein was pre-incubated with a series of dilution of compounds in the reaction buffer (40 mM Tris [pH 7.5], 20 mM MgCl_2_, and 0.1 mg/ml BSA), at 25°C for 30 min. Then 3 μM ATP was added for another incubation of 30 min at 25°C, and Kinase-Glo™ Reagent was added to detect the rest amount of ATP, as recorded from luminescence measurements (RLU). In parallel, theYycG' protein with no addition of compounds was used as the control. The rate of inhibiting protein phosphorylation (R_p_) by the compounds was calculated from equation 1:

Rp=RLU(YycG′+compound+ATP+Reagent)−RLU(YycG′+ATP+Reagent)RLU(ATP+Reagent)−RLU(YycG′+ATP+Reagent)×100%     (1)
 MathType@MTEF@5@5@+=feaafiart1ev1aaatCvAUfKttLearuWrP9MDH5MBPbIqV92AaeXatLxBI9gBaebbnrfifHhDYfgasaacH8akY=wiFfYdH8Gipec8Eeeu0xXdbba9frFj0=OqFfea0dXdd9vqai=hGuQ8kuc9pgc9s8qqaq=dirpe0xb9q8qiLsFr0=vr0=vr0dc8meaabaqaciaacaGaaeqabaqabeGadaaakeaacqqGsbGudaWgaaWcbaGaeeiCaahabeaakiabg2da9maalaaabaGaeeOuaiLaeeitaWKaeeyvauLaeiikaGIaeeywaKLaeeyEaKNaee4yamMafe4raCKbauaacqGHRaWkcqqGJbWycqqGVbWBcqqGTbqBcqqGWbaCcqqGVbWBcqqG1bqDcqqGUbGBcqqGKbazcqGHRaWkcqqGbbqqcqqGubavcqqGqbaucqGHRaWkcqqGsbGucqqGLbqzcqqGHbqycqqGNbWzcqqGLbqzcqqGUbGBcqqG0baDcqGGPaqkcqGHsislcqqGsbGucqqGmbatcqqGvbqvcqGGOaakcqqGzbqwcqqG5bqEcqqGJbWycuqGhbWrgaqbaiabgUcaRiabbgeabjabbsfaujabbcfaqjabgUcaRiabbkfasjabbwgaLjabbggaHjabbEgaNjabbwgaLjabb6gaUjabbsha0jabcMcaPaqaaiabbkfasjabbYeamjabbwfavjabcIcaOiabbgeabjabbsfaujabbcfaqjabgUcaRiabbkfasjabbwgaLjabbggaHjabbEgaNjabbwgaLjabb6gaUjabbsha0jabcMcaPiabgkHiTiabbkfasjabbYeamjabbwfavjabcIcaOiabbMfazjabbMha5jabbogaJjqbbEeahzaafaGaey4kaSIaeeyqaeKaeeivaqLaeeiuaaLaey4kaSIaeeOuaiLaeeyzauMaeeyyaeMaee4zaCMaeeyzauMaeeOBa4MaeeiDaqNaeiykaKcaaiabgEna0kabigdaXiabicdaWiabicdaWiabcwcaLiaaxMaacaWLjaWaaeWaaeaacqaIXaqmaiaawIcacaGLPaaaaaa@A46C@

IC_50 _(the concentration of inhibition of 50% YycG' protein autophosphorylation) was obtained by using the Origin v7.0 software (OriginLab, Northampton, USA).

### Cytotoxicity and erythrocyte hemolysis assays

Cytotoxicity of the antibacterial compounds on cultured Vero cell was measured by using the Cell Proliferation Kit I (MTT) (Roche, Indianapolis, USA) according to the manufacturer's protocol. Addition of DMSO (1%) in the medium produced a slight cytotoxicity on Vero cell, which could easily be corrected for. Each assay was performed in quadruplicate and repeated three times. The results were converted to percentage of the control (cells only treated with 1% DMSO) and CC_50 _(concentrations that produce a 50% cytotoxicity effect on Vero cell) was calculated by the Origin v7.0 software (OriginLab, Northampton, USA). Hemolytic activities of the compounds were determined by using healthy human erythrocytes [61]. The erythrocytes were washed three times in sterile saline and resuspended to 5% prior to the assay. Then a volume of 200 μl cell suspension containing MIC or 4×MIC concentrations of the compounds was added in quadruplicate to the wells of 96-well microtiter plates (Falcon). Cells without compound treatment and cells with 1% Triton-100 treatment were used as 0% and 100% hemolysis controls, respectively. The cell suspensions were incubated for 1 hour at 37°C and centrifuged at 1000×g for 10 min. Volumes of 100 μl supernatants were transferred to another sterile plate and hemoglobin release from the cells was determined at 570 nm. Addition of DMSO (1%) in the medium did not affect the integrity of erythrocyte membrane. The hemolysis assays were repeated twice.

### Pharmacophore model building

The best conformations of the six inhibitor outputs from Dock4.0 were superimposed using the DISCO program [62] encoded in Sybyl6.8. Multi-conformations were generated for the Flex Searches using Multisearch and REJECT features. The REJECT feature removes duplicates to leave a set of unique low energy conformers. DISCO produces a number of possible pharmacophores. The hypotheses were grouped on the basis of the assignment of atoms to features. Four features containing two hydrogen donor and two hydrophobic centers were detected by DISCO.

## Authors' contributions

SM, AD, HJ and DQ conceived and designed the whole project. JZ performed the modeling of YycG protein and structure-based virtual screening for potential inhibitors, under the supervision of HJ. ZQ, BX, LC, YW and XY participated in the biological experiments about lead-compounds, XS, SM and DQ supervised the experiments. ZQ, JZ, LC, YW and AD analyzed the data and produced figures. ZQ, JZ, SM, AD, HJ, and DQ drafted the manuscript. All the authors have read and approved the final manuscript.

## Supplementary Material

Additional File 1 **Expression and purification of the recombinant YycG' protein**. SDS-PAGE analysis of crude extracts from *E. coli *BL21 carrying pET28a (lane 2, prior to IPTG induction; lane 3, IPTG induction), pETYycG' (lane 4, prior to IPTG induction; lane 5, IPTG induction), purified YycG' (lane 6, approximately 34 kDa), and molecular weight standards were loaded in lane 1. Click here for file

Additional File 2 **Measurement of kinase activity of YycG' protein *in vitro***. Luminescent output correlates with amount of ATP (A). A direct relationship exists between the luminescence measured with the Kinase-Glo™ Reagent and the amount of ATP. A constant amount of YycG' protein (4 μg) was added into reaction systems containing variant ATP concentrations (B). The respective reaction system without YycG' treatment was used as control. Variant amounts of YycG' protein was added into reaction systems containing a constant ATP concentration (50 μM). Each assay was performed in quadruplicate and repeated three times. The values represented the mean and SD of one separate experiment. Click here for file

Additional File 3 **Comparison of inhibiting protein autophosphorylation of YycG' and SrrB' by 6 potential YycG inhibitors**. All the compounds were used at the concentration of 50 μM, and each reaction system contained 4 μg purified protein and 3 μM ATP (see Methods). Click here for file

## Figures and Tables

**Table 1:** Minimal inhibitory concentrations and minimal bactericidal concentrations of seven potential inhibitors of the YycG histidine kinase

Chemical inhibitor ^a^	Minimal inhibitory concentration (MIC, μM)					Minimal bactericidal concentration (MBC, μM)	
	
	*S. epidermidis* ATCC12228	*S. epidermidis* RP62A	*S. aureus* ATCC29213	*S. pyogenes* *144*	*S. mutans* *128*	*S. epidermidis* ATCC12228	*S. epidermidis* RP62A
**Compound 1**	50	50	50	200	>200	200	200
**Compound 2**	25	25	25	100	100	100	100
**Compound 3**	12.5	25	100	25	>200	50	100
**Compound 4**	12.5	12.5	>200	50	>200	50	100
**Compound 5**	6.25	6.25	6.25	1.56	6.25	25	25
**Compound 6**	100	100	>200	undone	undone	undone	undone
**Compound 7**	0.2	>200	>200	>200	>200	>400	>400

^a ^Stock solution of the compounds were prepared in dimethyl sulfoxide (DMSO) at the concentration of 200 μM. The molecule weights of these seven compounds are: compound **1 **(652), compound **2 **(519), compound **3 **(532), compound **4 **(440), compound **5 **(623), compound **6 **(555) and compound **7 **(505).

**Table 2:** Biological effects of seven potential inhibitors of the YycG histidine kinase

Chemical inhibitors	MICs (μM) ^a^	*K*_*D *_value (μM) ^b^	IC_50 _(μM) for YycG' ^c^	CC_50 _(μM) on Vero cell ^d^	Hemolysis (%) ^e^
**Compound 1**	50	27.8	48	>200	< 0.1 (< 0.1)
**Compound 2**	25	11.1	29	96	2.3 (6.1)
**Compound 3**	12.5	40.4	15	152	< 0.1 (0.5)
**Compound 4**	12.5	2.8	13.5	>200	0.1 (0.4)
**Compound 5**	6.25	2.3	14	>200	0.1 (0.3)
**Compound 6**	100	--	>200	>200	1.1 (2.3)
**Compound 7**	0.2	15.7	6.5	>200	< 0.1 (< 0.1)

^a ^The concentrations (μM) listed here were equal to those listed in Table 2, as tested on *S. epidermidis *ATCC 12228.

^b ^*K*_*D *_value represents the binding affinity of various inhibitors to the YycG' protein, compound **6 **showed a very low binding affinity, and could not calculate its *K*_*D *_value with software.

^c ^IC_50 _represents the concentration of inhibition of 50% the YycG' protein autophosphorylation. As the highest concentration tested was 50 μM, compound **6 **displayed poor ability of inhibition.

^d ^CC_50 _represents the concentration that produce a 50% cytotoxicity effect on Vero cell, as the highest concentration tested corresponding to 200 μM.

^e ^The healthy human erythrocytes were used for the hemolysis assay, and the hemolytic activity of seven inhibitors were shown at their MICs and 4 × MICs (the numbers in the parenthesis) for *S. epidermidis *ATCC12228 strain.
