# Supplementary figures and images for: Structure-based discovery of inhibitors of the YycG histidine kinase: New chemical leads to combat Staphylococcus epidermidis infections
Source: BMC Microbiol. 2006 Nov 10;6:96. doi: 10.1186/1471-2180-6-96 (PMC1660542; doi:10.1186/1471-2180-6-96)

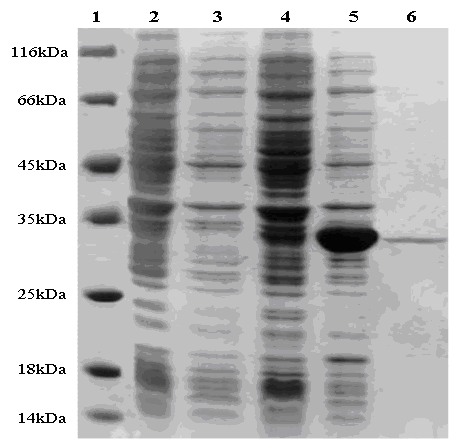

Supplement: Additional File 1 — Expression and purification of the recombinant YycG' protein. SDS-PAGE analysis of crude extracts from E. coli BL21 carrying pET28a (lane 2, prior to IPTG induction; lane 3, IPTG induction), pETYycG' (lane 4, prior to IPTG induction; lane 5, IPTG induction), purified YycG' (lane 6, approximately 34 kDa), and molecular weight standards were loaded in lane 1. [file 1471-2180-6-96-S1.tiff]

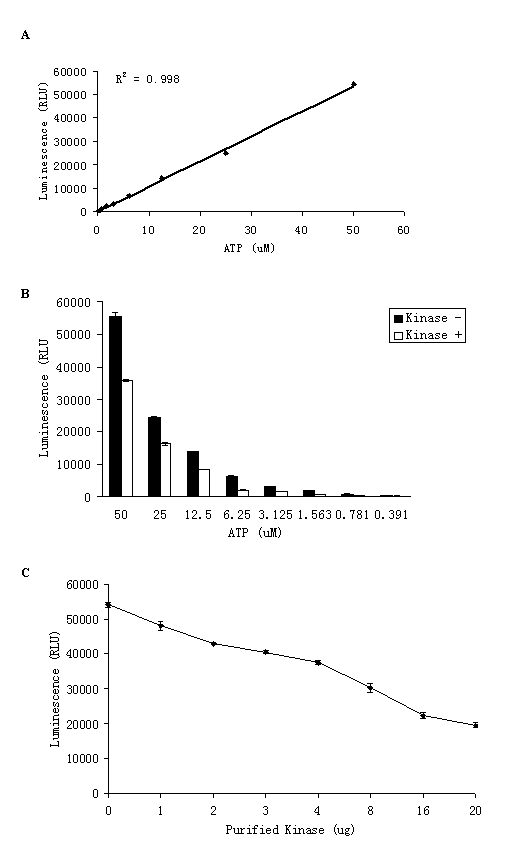

Supplement: Additional File 2 — Measurement of kinase activity of YycG' protein in vitro. Luminescent output correlates with amount of ATP (A). A direct relationship exists between the luminescence measured with the Kinase-Glo™ Reagent and the amount of ATP. A constant amount of YycG' protein (4 μg) was added into reaction systems containing variant ATP concentrations (B). The respective reaction system without YycG' treatment was used as control. Variant amounts of YycG' protein was added into reaction systems containing a constant ATP concentration (50 μM). Each assay was performed in quadruplicate and repeated three times. The values represented the mean and SD of one separate experiment. [file 1471-2180-6-96-S2.tiff]
