# Supplementary material for: Structure-based discovery of inhibitors of the YycG histidine kinase: New chemical leads to combat Staphylococcus epidermidis infections
Source: BMC Microbiol. 2006 Nov 10;6:96. doi: 10.1186/1471-2180-6-96 (PMC1660542; doi:10.1186/1471-2180-6-96)
Supplement: Additional File 3 — Comparison of inhibiting protein autophosphorylation of YycG' and SrrB' by 6 potential YycG inhibitors. All the compounds were used at the concentration of 50 μM, and each reaction system contained 4 μg purified protein and 3 μM ATP (see Methods). [file 1471-2180-6-96-S3.doc]

**Additional File 1. Comparison of inhibiting protein autophosphorylation of YycG’ and SrrB’ by 6 potential YycG inhibitors ^a^**

|  | Inhibition of YycG’ phosphorylation | Inhibition of SrrB’ phosphorylation |
| --- | --- | --- |
| **Compound 1** | 52% | 3% |
| **Compound 2** | 61% | 3% |
| **Compound 3** | 70% | 5% |
| **Compound 4** | 76% | 3% |
| **Compound 5** | 73% | 20% |
| **Compound 7** | 86% | 2% |

^a^ All the compounds were used at the concentration of 50 μM, and each reaction system contained 4 μg purified protein and 3 μM ATP (see Materials and Methods).
